# Two homologous host proteins interact with potato virus X RNAs and CPs and affect viral replication and movement

**DOI:** 10.1038/srep28743

**Published:** 2016-06-29

**Authors:** Hoseong Choi, Won Kyong Cho, Kook-Hyung Kim

**Affiliations:** 1 Department of Agricultural Biotechnology, Seoul National University, Seoul, Korea; 2 Research Institute of Agriculture and Life Sciences, Seoul National University, Seoul, Korea; 3 Plant Genomics and Breeding Institute, Seoul National University, Seoul, Korea

## Abstract

Because viruses encode only a small number of proteins, all steps of virus infection rely on specific interactions between viruses and hosts. We previously screened several *Nicotiana benthamiana* (Nb) proteins that interact with the stem-loop 1 (SL1) RNA structure located at the 5′ end of the potato virus X (PVX) genome. In this study, we characterized two of these proteins (NbCPIP2a and NbCPIP2b), which are homologous and are induced upon PVX infection. Electrophoretic mobility shift assay confirmed that both proteins bind to either SL1(+) or SL1(−) RNAs of PVX. The two proteins also interact with the PVX capsid protein (CP) *in planta*. Overexpression of NbCPIP2a positively regulated systemic movement of PVX in *N. benthamiana,* whereas NbCPIP2b overexpression did not affect systemic movement of PVX. Transient overexpression and silencing experiments demonstrated that NbCPIP2a and NbCPIP2b are positive regulators of PVX replication and that the effect on replication was greater for NbCPIP2a than for NbCPIP2b. Although these two host proteins are associated with plasma membranes, PVX infection did not affect their subcellular localization. Taken together, these results indicate that NbCPIP2a and NbCPIP2b specifically bind to PVX SL1 RNAs as well as to CP and enhance PVX replication and movement.

*Potato virus X* (PVX), which belongs to the genus *Potexvirus*, is an important pathogen of potato and other solanaceous plants and is considered one of the 10 most important plant viruses^1^,^2^,^3^. The PVX genome, which is approximately 6.4 kb long and is capped and polyadenylated, is composed of a 84-nucleotide (nt) 5′-nontranslated region (NTR), five viral open reading frames (ORFs), and a 72-nt 3′-NTR^4^. The five ORFs encode an RNA-dependent RNA polymerase (RdRp), triple gene blocks proteins (TGBs), and the capsid protein (CP). In general, RNA elements at these NTRs of RNA viruses include sequences and structural elements important for viral translation and replication; they also function as binding sites for cellular proteins required for maintaining the viral infection cycle^5^,^6^,^7^. The 5′ NTR of the PVX genome contains five repeated ACCA sequences followed by three stem-loop structures (5′ SL1, 5′ SL2, and 5′ SL3), while the 3′ NTR contains three stem-loop structures (3′ SL1, 3′ SL2, and 3′ SL3) and a poly(A) tail^8^,^9^. The 5′ SL1 is particularly important for PVX translation, viral RNA replication, and initiation of PVX virion assembly^2^,^5^,^10^. The CP of PVX also plays an important role during virus encapsidation and movement^11^,^12^.

Viruses encode only a few proteins, and virus replication and pathogenicity therefore depend on specific interactions between viral elements and host factors^13^,^14^,^15^. Many host factors that interact with viral RNAs and proteins have been identified by yeast-two hybrid (Y2H) assay, bimolecular fluorescence complementation (BiFC), co-immunoprecipitation (Co-IP), and Northwestern blot analysis followed by matrix-assisted laser desorption ionization time-of-flight mass spectrometry (MALDI TOP MS). Several host proteins have been identified that interact with PVX viral proteins including NbPCIP1, TIP, NbMPB2Cb, NbDnaJ, eEF1A, and eEF1Bβ^16^,^17^,^18^,^19^,^20^. Moreover, researchers have used Northwestern blot followed by MALDI TOP MS to identify tobacco proteins that bind to PVX 5′ SL RNAs^21^,^22^.

The functions of several host proteins in PVX infection have been determined. For example, TGB12K interacting proteins (TIP1 to TIP3) specifically interact with TGB2 protein and are associated with callose degradation and cell-to-cell movement of PVX^18^. The interaction between NbPCIP1 and CP affects PVX replication^16^. NbMPB2Cb interacts with four PVX viral proteins as well as with SL1 RNAs and reduces PVX movement^19^. The binding of NbDnaJ to minus-strand SL1 RNA reduces PVX replication and movement^20^. Interaction between the WRKY1 transcription factor with SL1 is important for viral RNA accumulation and disease symptom development^22^. The interaction between eukaryotic translation elongation factor 1 (eEF1) and TGB1 protein facilitates PVX infection^17^.

In this study, we characterized two homologous host proteins that were previously determined to interact with SL RNAs of PVX^21^. Electrophoretic mobility shift assay (EMSA) as well as BiFC were used to confirm the interactions of host proteins and PVX viral components. The effects of these host proteins on PVX replication and movement were determined with transient overexpression and silencing experiments.

## Results

### Characterization of NbCPIP2a and NbCPIP2b interacting with SL1 RNA structures of PVX

**Figure 1:**
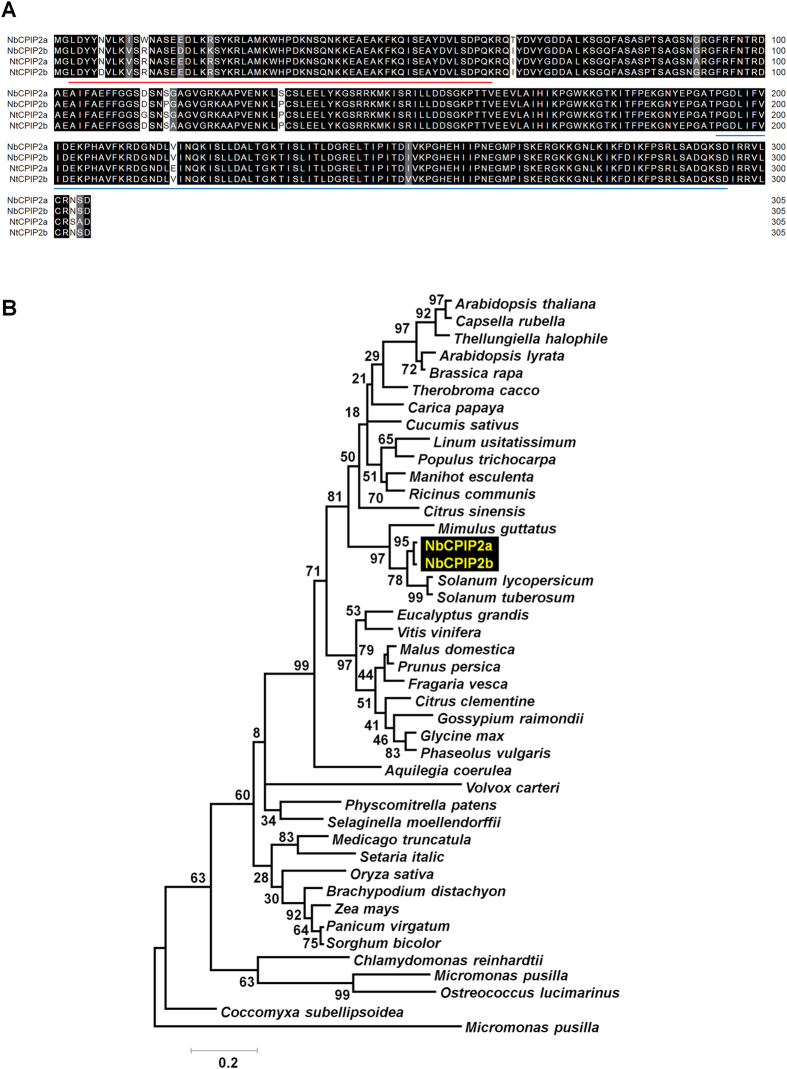
Sequence alignment of NbCPIP2a, NbCPIP2b, NtCPIP2a, and NtCPIP2b proteins and phylogenetic analysis of NbCPIP2a-like proteins. (**A**) Abbreviations for gene names are as follows: Nb2a (NbCPIP2a), Nb2b (NbCPIP2b), Nt2a (NtCPIP2a), and Nt2b (NtCPIP2b). The red line and blue line indicate the conserved DnaJ domain and the DnaJ C terminal domain, respectively. Aligned sequences were visualized with the Megalign program. (**B**) Phylogenetic relationship of NbCPIP2a-like proteins identified from plants for which draft genome sequences are available. The phylogenetic tree was constructed by protein sequences of NbCPIP2a-like proteins from diverse plants. NbCPIP2a-like proteins were identified by blast search in the Phytozome database. For simplicity, only plant species names instead of protein names are indicated in the phylogenetic tree. The phylogenetic tree was generated by the neighbor-joining method with 1,000 bootstrap replicates and Kimura 2-parameter distance using the MEGA6 program^55^.

As noted, we previously detected 24 *N. benthamiana* proteins that interact with SL1 RNAs of PVX^21^. Of the 24 proteins, we selected two that are homologous to known potyviral CP interacting protein 2a (CPIP2a) from *N. tabacum*^23^. A blast search using CPIP2a protein as a query against available draft genomes for *N. benthamiana* and *N. tabacum* revealed that each *Nicotiana* genome contains two homologues of these proteins designated as NbCPIP2a and NbCPIP2b. We found that these two proteins are about 34 kDa in size (305 amino acids) and contain two conserved DnaJ domains. We compared the protein sequences of NbCPIP2a and NbCPIP2b as well as of NtCPIP2a and NtCPIP2b. The amino acid sequences of these four proteins differ in only 11 amino acids (Fig. 1A). NbCPIP2 homologous proteins were searched against the Phytozome database to determine the evolutionary origin and divergence of NbCPIP2 in plants. A phylogenetic tree using NtCPIP2a homologs showed that NbCPIP2a and NbCPIP2b were grouped with members of the *Solanaceae* such as *Solanum lycopersicum* and *S. tuberosum* (Fig. 1B). Interestingly, an NbCPIP2a orthologue was also present in marine algae *Coccomyxa subellipsoidea* and *Micromonas pusilla*.

To confirm the interaction between NbCPIP2a and NbCPIP2b with SL1 RNAs of PVX, we conducted EMSA^24^. The 5′ SL1(−) RNA transcripts generated shifted complexes when incubated with the NbCPIP2a and NbCPIP2b in EMSA and the abundance of the shifted complexes increased as the abundance of RNA transcripts increased (Supplementary Figure S1). RNA transcript containing 5′ SL1(+) bound only to NbCPIP2b (Supplementary Figure S1). Moreover, both host proteins can bind to SL structures at the 3′ SLs of PVX RNA. This result indicates that the two homologous proteins might differ in their binding to the SL RNA structures of PVX. Overall, RNA transcripts containing PVX 5′ SL1 or 3′ SLs were capable of forming RNA–protein complexes with two homologous tobacco proteins, NbCPIP2a and NbCPIP2b.

### NbCPIP2a and NbCPIP2b also interact with PVX CP

**Figure 2:**
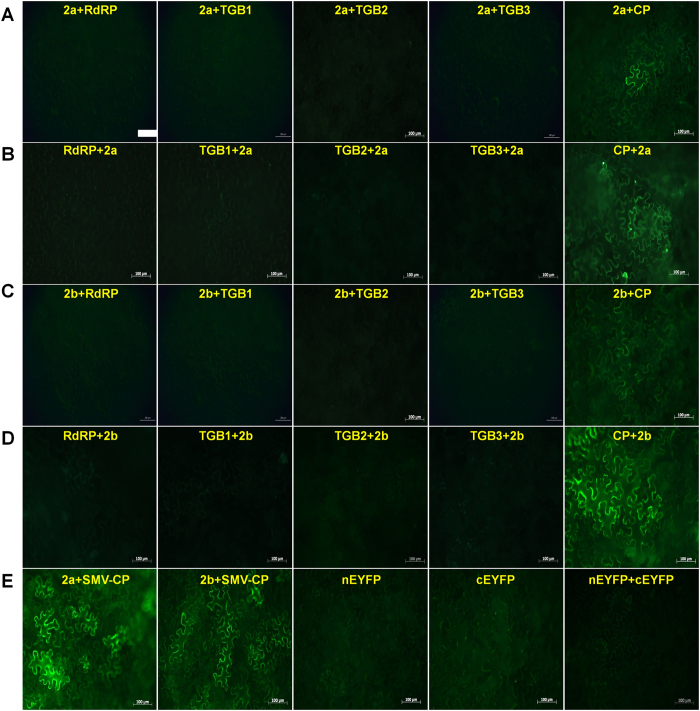
Interactions between identified host proteins and PVX viral proteins. To study protein-protein interactions, we used the BiFC system *in planta*. Two different sets of vectors containing split YFP were used. (**A**) Interaction of NbCPIP2a fused to nYFP with each viral protein fused to cYFP and (**B**) vice versa. (**C**) Interaction of NbCPIP2b fused to nYFP with each viral protein fused to cYFP, and (**D**) vice versa. (**E**) Positive controls, including the interaction of NbCPIP2a or NbCPIP2b fused to nYFP with SMV CP fused to cYFP. Negative controls, including nEYFP, cEYFP, and both nEYFP and cEYFP. The white bar indicates 100 μm.

To determine whether NbCPIP2a and NbCPIP2b directly interact with PVX proteins including RdRp, TGB1, TGB2, TGB3, and CP, we carried out an *in planta* BiFC assay using the *Agrobacterium*-infiltration method^4^. In the BiFC assay, we used divided yellow fluorescence protein (YFP), referred to as YFP-N (N-terminal) and YFP-C (C-terminal). Because the different combinations of YFP-fused proteins might affect the protein-protein interaction^25^, we generated two different sets of YFP fusion constructs (Fig. 2). For example, host proteins were fused to YFP-N and viral proteins were fused to YFP-C (Fig. 2A,C), and vice versa (Fig. 2B,D). The combination of negative controls (nEYFP, cEYFP, and nEYFP + cEYFP) did not produce any fluorescence. The BiFC assay demonstrated that NbCPIP2a and NbCPIP2b interact with CP but not with the other four PVX proteins (Fig. 2A–D).

A previous report indicated that the CP of *Potato virus Y* (PVY), which is a *Potyvirus*, interacts with NtCPIP2a and NtCPIP2b^23^. Therefore, we hypothesized that NbCPIP2 can interact with the CPs of other potyviruses. We tested this hypothesis with CPs of the following potyviruses: soybean mosaic virus (SMV), pepper mottle virus (PepMoV), pepper severe mosaic virus, potato virus A (PVA), PVY, turnip mosaic virus (TuMV), and zucchini yellow mosaic virus. The CP of SMV interacted with both NbCPIP2a and NbCPIP2b (Fig. 2E). Although NbCPIP2b bound to the CP of PepMoV, PVA, and TuMV, NbCPIP2a bound only to the CP of TuMV (Supplementary Figure S2). In addition, we found that the interaction between host protein:YFP-C and viral protein:YFP-N was much stronger than the interaction between host protein:YFP-N and viral protein:YFP-C.

### NbCPIP2a and NbCPIP2b enhance PVX RNA replication

**Figure 3:**
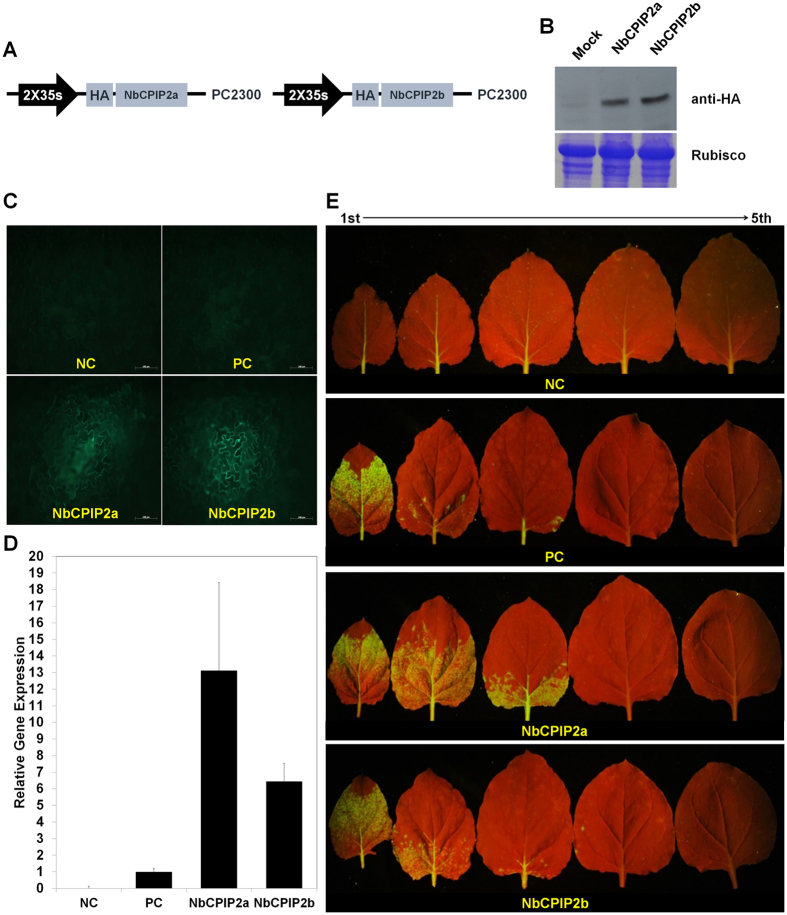
Transient overexpression of NbCPIP2a and NbCPIP2b in response to PVX infection. (**A**) Schemes of overexpression vectors. (**B**) Western blot analysis to confirm transient overexpression of the two host proteins in *N. benthamiana*. (**C**) Intercellular movement of GFP-tagged PVX in the transiently overexpressed *N. benthamiana* leaves. (**D**) PVX viral RNA replication in the extracted protoplasts. (**E**) Long-distance movement of GFP-tagged PVX in the transiently overexpressed *N. benthamiana* leaves. NC = negative control (leaves were not inoculated with overexpression vectors or with PVX). PC = positive control (leaves were not inoculated with overexpression vectors but were inoculated with PVX). 1^st^ = the first leaf from the top. 5^th^ = the fifth leaf from the top. NbCPIP2a or NbCPIP2b (leaves were inoculated with overexpression vectors of NbCPIP2a or NbCPIP2b and were also inoculated with PVX).

To characterize the functions of the two host proteins in response to PVX infection, we generated vectors for the transient overexpression and silencing of the two genes that encode the proteins. For transient overexpression, we generated vectors containing dual 35S promoters of cauliflower mosaic virus and tagged each host gene with hemagglutinin epitope (HA-tag) (Fig. 3A). At 2 days after infiltration, we extracted total proteins from the *N. benthamiana* leaves that were infiltrated with the overexpression vector. Western blot analyses using anti-HA confirmed transient overexpression of the two host proteins in *N. benthamiana* (Fig. 3B). After transient overexpression of each gene, we inoculated the leaves with PVX tagged with green fluorescence protein (GFP). The area with green fluorescence was much wider on the leaves that transiently overexpressed NbCPIP2a or NbCPIP2b than on the leaves that were inoculated only with PVX (the positive control). Movement of GFP-tagged PVX was faster with transient overexpression of NbCPIP2a than with transient overexpression of NbCPIP2b (Fig. 3C).

Next, we examined PVX replication in *N. benthamiana* protoplasts. We first transiently overexpressed each gene in *N. benthamiana* leaves. On the next day, we inoculated the leaves with PVX by agro-infiltration. We prepared protoplasts after 12 h, and incubated the protoplasts at 25 °C in a growth chamber for 2 days. We then extracted total RNAs from the protoplasts according to a previous study^26^. Real-time RT-PCR results showed that PVX replication was about 13-times and 6.5-times higher in the protoplasts overexpressing NbCPIP2a and NbCPIP2b, respectively, than in the positive control (Fig. 3D).

For systemic movement assay, we transiently overexpressed NbCPIP2a and NbCPIP2b, respectively, on *N. benthamiana* leaf. The fourth leaves from the top at a 5–6 leaves stage *N. benthamiana* were agro-infiltrated. Two days later, we agro-infiltrated GFP-tagged PVX in the same leaf which overexpressing individual host protein. At 7 days after inoculation of GFP-tagged PVX, we examined the green fluorescence in the upper leaves. In the positive control, only the first leaf from the top showed green fluorescence. Green fluorescence was evident in the first, second, and third leaves from the top in plant which was infiltrated with NbCPIP2a in local leaf. However, we observed green fluorescence in the first and second leaves from the top in the plant which was infiltrated with NbCPIP2b in local leaf (Fig. 3E).

**Figure 4:**
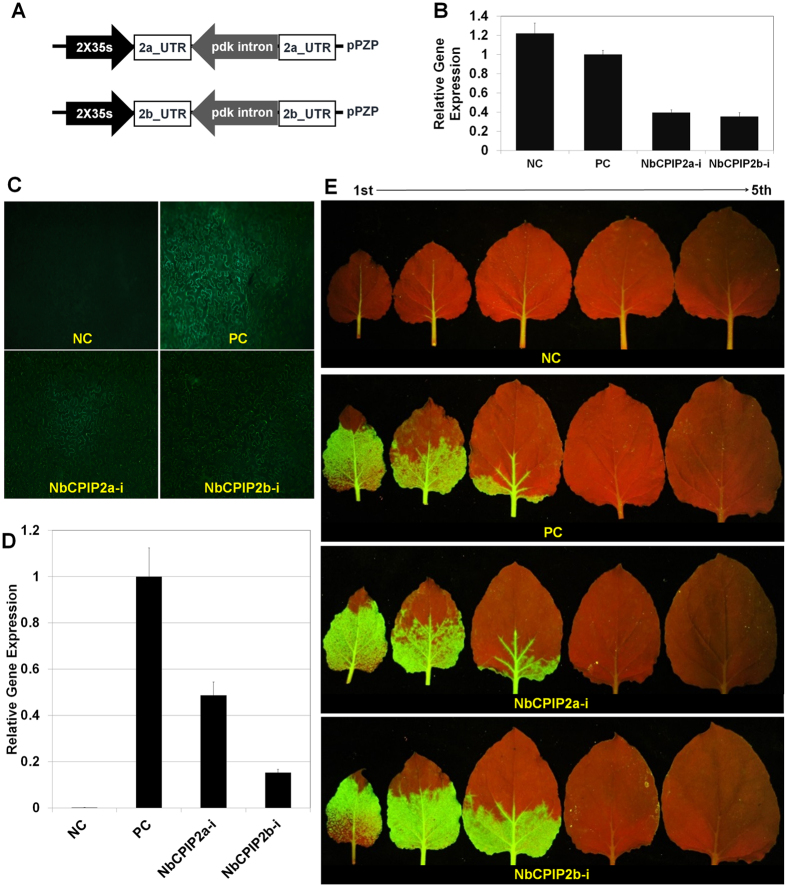
Transient silencing of NbCPIP2a and NbCPIP2b in response to PVX infection. (**A**) Schemes of silencing vectors. (**B**) Gene-silencing activities induced by RNAi vectors. (**C**) Intercellular movement of GFP-tagged PVX in transiently silenced *N. benthamiana* leaves. (**D**) PVX viral RNA replication in the extracted protoplasts. (**E**) Long-distance movement of GFP-tagged PVX in the transiently silenced *N. benthamiana* leaves. NC = negative control (leaves were not silenced and were not inoculated with PVX). PC = positive control (leaves were not silenced but were inoculated with PVX). 1^st^ = the first leaf of the top. 5^th^ = the fifth leaf from the top. NbCPIP2a-i or NbCPIP2b-i (leaves were infiltrated with the silencing vectors of NbCPIP2a or NbCPIP2b and were inoculated with PVX).

For transient silencing of the two host genes, we generated RNAi silencing vectors (Fig. 4A). To verify the silencing of target gene expression, we carried out real-time RT-PCR using gene-specific primers (Supplementary Table S1). After normalization based on expression of *actin* and *ubiquitin* genes, we calculated relative expression for *NbCPIP2a* and *NbCPIP2b* in the silenced leaves. Relative to their expression in the negative control leaves (i.e., leaves that were not silenced and were not inoculated with PVX), *NbCPIP2a* expression was reduced by 32.3% and *NbCPIP2b* expression was reduced by 28.9% in the silenced leaves (Fig. 4B). After transient silencing of each gene, we performed a challenge inoculation of GFP-tagged PVX on the inoculated leaves using agrobacterium. The area with green fluorescence and the intensity of the fluorescence were much less on the inoculated leaves where *NbCPIP2a* or *NbCPIP2b* was transiently silenced than on the non-silenced, positive control leaves, which were only inoculated with GFP-tagged PVX (Fig. 4C). We then examined PVX replication in the protoplasts extracted from silenced leaves for each gene. PVX replication was reduced by 48.4% in the *NbCPIP2a*-silenced leaves and by 15.1% in the *NbCPIP2b*-silenced leaves (Fig. 4D). We again examined the long-distance movement of GFP-tagged PVX in the transiently silenced *N. benthamiana*. Unexpectedly, PVX long-distance movement was similar in the silenced leaves and the positive control leaves (Fig. 4E).

### PVX infection does not alter the subcellular localization of NbCPIP2a or NbCPIP2b

**Figure 5:**
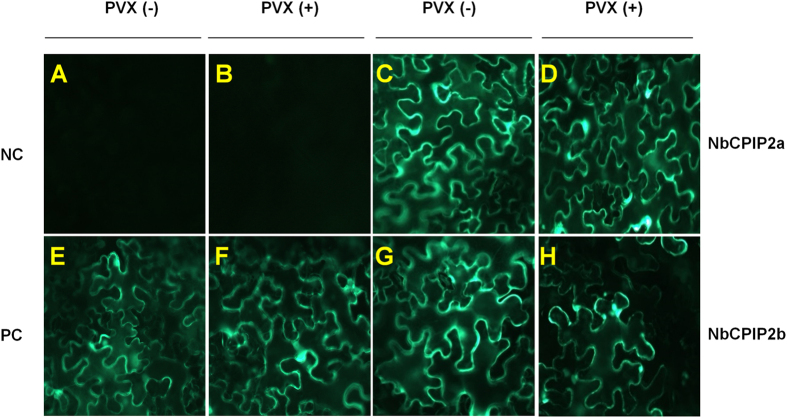
Subcellular localization of the two host proteins in response to PVX infection. (**A,B,E,F**) Leaves were not infiltrated with GFP-tagged expression vectors for NbCPIP2a or NbCPIP2b and were inoculated (**B,F**) or were not inoculated (**A,E**) with PVX. (**C,D,G,H**) Leaves were infiltrated with GFP-tagged expression vectors for NbCPIP2a (**C,D**) or NbCPIP2b (**G,H**) and were inoculated (**D,H**) or were not inoculated (**C,G**) with PVX.

To determine the subcellular localization of NbCPIP2a and NbCPIP2b, we infiltrated the GFP-tagged expression vector for each gene; green fluorescence for the two proteins was mostly evident in the plasma membranes (Fig. 5C,G). We then examined the subcellular localization of the two proteins following PVX infection; green fluorescence for the two proteins was once again mostly evident in the plasma membranes (Fig. 5D,H). Free GFP was dispersed in most cellular organelles. As compared to free GFP, we did not observe change of subcellular localization for two host proteins after PVX infection. This result indicates that PVX infection does not affect the subcellular localization of NbCPIP2a or NbCPIP2b.

### *NbCPIP2* expression is induced by PVX infection but not by CMV or PepMoV infection

**Figure 6:**
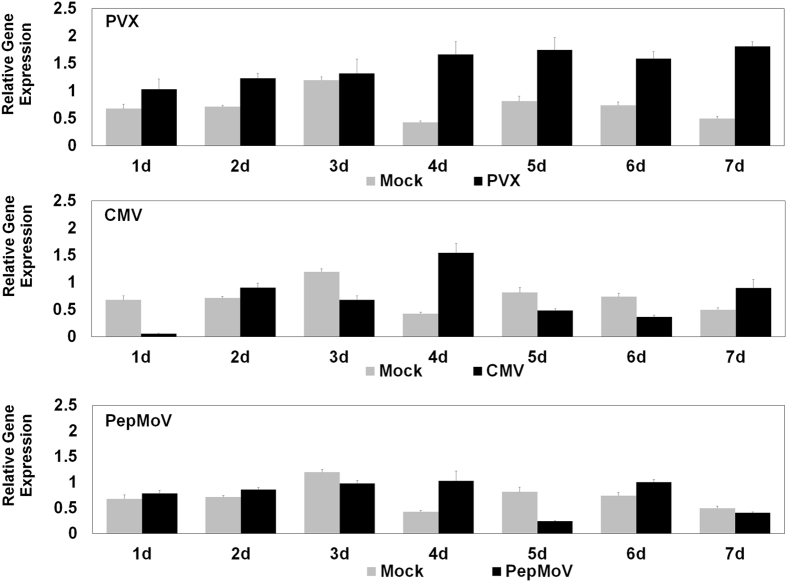
Time-course analysis of host gene expression in response to PVX, CMV, and PepMoV infection. *N. benthamiana* leaves were inoculated with the indicated virus or were not inoculated (mock). Leaf samples were collected 1 to 7 days later, and expression of *NbCPIP2a* or *NbCPIP2b* was quantified. For each combination of treatment and time, three independent plants were sampled. Expression of NbCPIP was normalized based on expression of ubiquitin and actin genes. Values are means + SD.

To monitor the expression of *NbCPIP* genes over time in response to infection by PVX, cucumber mosaic virus (CMV), and PepMoV, we conducted real-time RT-PCR analysis using a primer-pair that amplifies both genes (Fig. 6). The expression of *NbCPIP2* genes increased steadily for the first 4 days and remained high for the next 3 days in plants inoculated with PVX but not in plants inoculated with CMV or PepMoV (Fig. 6). Expression of *NbCPIP2* genes in non-inoculated plants tended to increase for the first 3 days but then did not exhibit a clear pattern. These results suggest that the expression level of NbCPIP2 might be regulated by specific viruses.

## Discussion

Because viruses have small genomes that encode only a few proteins, they rely on host factors for their replication, movement, and pathogenicity^15^,^27^. To identify such host factors, researchers have often investigated the interactions between host proteins and virus proteins or between host proteins and viral RNAs. In the current study, we characterized two homologous host proteins that interact with PVX SL1 RNAs and CP.

A previous study characterized the functions of the host protein NtCPIP2a, which interacts with PVY CP in tobacco^23^. In the current study, we functionally characterized two NtCPIP2a-like proteins, NbCPIP2a and NbCPIP2b, in *N. benthamiana* in response to PVX infection. A phylogenetic analysis using NbCPIP2a-like proteins from diverse plants showed that NbCPIP2a-like proteins occur in most plant kingdoms, ranging from marine algae to higher plants. NbCPIP2a might have evolved from marine photosynthetic organisms. NbCPIP2a and NbCPIP2b have two conserved domains, i.e., the DnaJ domain (IPR001623) at the N-terminal and the HSP40/DnaJ peptide-binding domain (IPR008971) at the C-terminal regions^28^,^29^. Both domains occur in many prokaryotic and eukaryotic genomes. Researchers have often reported that HSP40/DnaJ is required for protein translation, folding/unfolding, trafficking, and secretion as co-chaperone of HSP70^28^,^30^. HSP70s and its co-chaperone HSP40/DnaJ are involved in virus replication^31^,^32^ and the assembly or disassembly of the virus capsid^33^. Specific binding between DnaJ/HSP40 proteins and other viral proteins has also been reported^23^,^34^.

Interactions between NbCPIP2a and NbCPIP2b with PVX SL1(+) RNAs have previously been shown by Northwestern blot followed by MS/MS analysis^21^. In contrast to the previous study, the current study demonstrated that NbCPIP2a interacts with SL1(−) but not with SL1(+) RNA. While the previous study identified a partial fragment of NbCPIP2a by MS/MS analysis, the current study used the complete cDNA sequence for cloning NbCPIP2a. Therefore, we believe that the results of the current study are more convincing than those of the previous study^21^. EMSA confirmed the different binding activities of two homologous proteins to PVX SL1 RNAs. We suspect that the difference in the five amino acids might explain the difference in the binding of the two host proteins to viral RNA. To our knowledge, this is the first report that demonstrates a difference in viral RNA-interaction capacity for two homologous proteins that differ in only five amino acids. The residue(s) within these host proteins that are responsible for controlling the interaction with PVX viral RNA structures should be determined in future research.

CPs of potyviruses are important for virion assembly, cell-to-cell movement, and long-distance movement^35^. To fulfill multiple functions in the host, CPs must interact with host components^36^. In this study the homologous host proteins have the same binding affinity for PVX CP; however, they differ in their interaction with SL1 RNAs of PVX. Furthermore, many homologous host proteins have a common conserved domain that is required for binding with CP. For example, a previous study used Y2H and BiFC to document that NtCPIP1 and NtCPIP2a interact with PVY CP^23^. In our BiFC assay, NbCPIP2a and NbCPIP2b interacted only with PVX CP but not with four other PVX proteins. Moreover, the binding to PVX CP did not differ between the two host proteins. NbCPIP2a and NbCPIP2b also interacted with the CPs of SMV, TuMV, PVA, and PepMoV, which belong to the genus *Potyvirus*. Although CPs of four potyviruses might interact with two host proteins, interaction ability of two host proteins were different based on BiFC results. For example, CPs of SMV and TuMV strongly interact with two host proteins. However, NbCPIP2a showed very weak interaction with CPs of PepMoV and PVA while NbCPIP2b displayed strong interaction with CPs of PepMoV and PVA. These results indicate binding abilities of two host proteins with potyviral CP might be virus specific. Based on these results, it is likely that CPs of potyviruses might have a conserved domain that is required for interaction with these two host proteins. A previous study using mutational analysis identified the CP core region (including the three highly conserved residues; S125, R157, D201 of PVY CP, which are essential for virion formation and plasmodesmal trafficking) as the interacting domain^23^,^35^.

The differences in the binding of the two host proteins to the PVX RNAs and CP suggested that the functions of the two host proteins might differ in response to PVX infection. To investigate that possibility, we performed transient overexpression and silencing experiments using *N. benthamiana*. Both host proteins were found to enhance PVX replication in experiments using protoplasts; the enhancement of PVX replication, however, was greater for NbCPIP2a than for NbCPIP2b. Long-distance movement PVX was also enhanced by both host proteins, and the effect of NbCPIP2a was slightly greater than that of NbCPIP2b. We also observed a decreased accumulation of PVX RNAs with partial silencing of the *NbCPIP2* genes, suggesting that NbCPIP2s are required during the initial stages of PVX RNA replication. Although overexpression of NbCPIP2a and NbCPIP2b increased RNA replication, cell-to-cell movement, and long-distance movement of PVX in *N. benthamiana*, partial silencing of these two genes reduced RNA replication but not cell-to-cell or long-distance movement of PVX. The intensity of fluorescence, however, was significantly reduced in silenced cells with respect to early cell-to-cell movement. This might result from the significantly reduced level of RNA replication during the early infection of silenced leaves. It is possible that 1) NbCPIP2s-mediated cell-to-cell movement of PVX is complemented by other host factor(s) in *N. benthamiana* or that 2) the silencing levels (32.3% and 28.9% for *NbCPIP2a* and *NbCPIP2b* genes, respectively) were too small to reduce the cell-to-cell movement of PVX. Regarding other host factors that may contribute to cell-to-cell movement, HSP70 proteins may enable viral movement complex to translocate through plasmodesmata (PD) pores^27^ and could provide the motive force that facilitates intercellular transport of plant virus via PD^37^. If both *NbCPIP2* genes are silenced, it is possible that HSP70 activity related to virus movement might be not affected because of functional complementation by DnaJ-like proteins that function as co-chaperones of HSP70; these include CPIP^23^, NtDNAJ-M541^37^, and RME-8^38^. Because HSP70 and its co-chaperone CPIP are required for PVY infection (presumably by interacting with the viral CP^23^), it is tempting to speculate that NbCPIP2a and NbCPIP2b act as important regulators during PVX infection by interacting with PVX SL1 RNA and CP and possibly by recruiting the HSP70. Therefore, characterization of DnaJ-like proteins involved in HSP70 interaction will increase our understanding of the roles of NbCPIP2a and NbCPIP2b in PVX replication and movement.

Several kinds of evidence indicate that the expression of *NbCPIP2s* might be specifically regulated by PVX and that NbCPIP2s might be important for PVX infection. First, the *NbCPIP2* genes were induced by PVX infection but not by CMV or PepMoV infection (Fig. 6). Second, the NbCPIP2s interacted with SL1 RNA elements of the PVX genomic RNA (gRNA) and CP. Third, over-expression of the NbCPIP2s significantly increased the accumulation of PVX RNAs and PVX movement (Fig. 3). Viral replicases of most plant viruses with plus-strand RNA associate with host cellular membranes by specific interaction with host proteins and form functional complexes for viral replication^39^,^40^,^41^,^42^. In PVX, RNA elements, including the first of five repeated ACCA sequence elements and the 5′ SL1 of PVX gRNA, are required for viral RNA replication and movement^5^,^43^,^44^,^45^,^46^. In addition, Lough *et al*.^47^ reported that the SL1(+) RNA or SL1(−) RNA of PVX behaves as a *cis*-acting element and can affect cell-to-cell movement of PVX. We recently identified host proteins, including NbMPB2Cb and NbDnaJ, that bind to the 5′ SL1 of PVX gRNA and reduce PVX RNA replication^19^,^20^. All of the identified host proteins, i.e., NbCPIP2s, NbMPB2Cb, and NbDnaJ, also interact with PVX CP, a protein that is also required for PVX movement. Moreover, subcellular localization of NbCPIP2s in host cellular membranes further indicates that NbCPIP2s may function in the formation of replicase complexes. Together, these results suggest that several host factors increase or decrease PVX RNA replication and movement possibly by the specifically interacting with an SL1 RNA element and by interacting with viral proteins such as CP or MP.

Overall, our study has demonstrated that the homologous host proteins NbCPIP2a and NbCPIP2b differ in their binding to PVX RNA elements but not to PVX CP. In addition, functional studies using transient assay showed that the difference in the binding properties of NbCPIP2a and NbCPIP2b resulted in functional differences, especially in PVX replication. We also found that the impaired functions of a host gene might be complemented by another homologous protein.

## Methods

### Plant materials and growth condition

*N. benthamiana* plants used in this study were grown in a growth chamber at 25 °C under a 16 h/8 h (light/dark) photoperiod. We used 4-week-old *N. benthamiana* plants for BiFC, transient expression, functional study, subcellular localization, and time-course gene expression analysis.

### RNA extraction and cloning of the two host genes

We extracted total RNAs from *N. benthamiana* plants with Isol-RNA Lysis Reagent (5 Prime, Hilden, Germany) according to the manufacturer’s instructions. Complementary DNA (cDNA) was synthesized with the GoScript Reverse Transcription System (Promega, Madison, USA) following the manufacturer’s instructions. Full-length *NbCPIP2a* and *NbCPIP2b* were amplified by gene-specific primers (Supplementary Table 1). The amplified PCR products were cloned into the Gateway pENTR vector following the manufacturer’s instructions (Invitrogen, Carlsbad, USA). The generated pENTR containing each gene was further cloned into appropriate vectors.

### Recombinant protein expression

Full-length *NbCPIP2a* and *NbCPIP2b* were cloned into the pET28 vector by restriction enzyme treatment using *Eco*RI and *Bam*HI. The cloned vectors were transformed into *E. coli* BL21(DE3) codon plus RIL strain (Stratagene, La Jolla, USA). Recombinant proteins were expressed with 0.4 mM IPTG at 37 °C and were extracted and then purified based on a previous study^47^. The purified proteins were subjected to EMSA.

### EMSA

Probes were amplified by PCR using probe-specific primers (Supplementary Table S1). After purification, *in vitro* transcription using T7 RNA polymerase (Takara, Shiga, Japan) and general procedures for EMSA were adapted from a previous study^48^. In brief, 100 ng of P^32^-labeled RNA transcripts and recombinant NbCPIP2a protein or NbCPIP2b protein were incubated on ice in 1X EMSA binding buffer (10 μl final volume). The mixtures were supplemented with a 0.2 volume of 5X loading buffer (50% glycerol and 0.05% bromophenol blue) and electrophoresed on a 5% non-denaturing polyacrylamide gel at 100 V (4 °C) for 40 min. The gels were dried with a vacuum dryer and visualized using a Fuji BAS-2500 Phosphor Imager (Fuji Film, Nakanuma, Japan).

### BiFC assay

The full-length of each gene in the pENTR vectors was cloned into the modified pPZP-DEST-nEYFP-C1 and pPZP-DEST-cEYFP-C1 vectors for BiFC^49^,^50^. The vectors containing NbCPIP2a and NbCPIP2b were transformed into *Agrobacterium* strain GV2260 by the heat shock method. The transformed bacteria were suspended in MMA buffer containing 10 mM MES salt (pH 5.6), 10 mM MgCl_2_ and 100 μM acetosyringone. A syringe was used to infiltrate the bacteria into the abaxial sides of *N. benthamiana* leaves. After 2 days, green fluorescence in the infiltrated leaves was observed with a fluorescence microscope (Carl Zeiss, Oberkochen, Germany).

### Transient overexpression and silencing of target host genes

To transiently overexpress the two host genes, we generated two overexpression constructs using pCAMBIA2300 vectors that were modified by tagging with the HA epitope. By *Stu*I enzyme digestion, each full-length cDNA for NbCPIP2a and NbCPIP2b was cloned into the modified pCAMBIA2300 vector. For viral replication and systemic movement experiment influenced by overexpressed NbCPIP2a and NbCPIP2b, we infiltrated vectors expressing NbCPIP2a and NbCPIP2b, respectively. Two days after transient overexpression of each protein, we inoculated the leaves with PVX tagged with green fluorescence protein (GFP) using an agrobacterium mediated infectious clone. As a viral replication observation in infiltrated leaves, we observed the PVX:GFP infiltrated leaves at 2 days after PVX:GFP infiltration. For systemic movement observation for PVX:GFP, we took pictures of PVX:GFP infected plants at 7 days after infiltration under hand UV-lamp. For transient silencing of the two host genes, a partial sequence for an individual host gene was cloned into a hairpin expression vector^51^, which was constructed by modifying the pPZP vector^16^. After four days infiltration of silencing agrobacterium clones, we analyzed the silencing level using total RNAs extracted from silenced leaves then inoculated PVX expressing GFP by agro-infiltration. We observed the viral replication in local leaves at 2 days post inoculation and systemic movement of PVX:GFP at 7 days post inoculation of PVX:GFP. All of cloned vectors were transformed into *Agrobacterium* strain GV3101 by the heat shock method. A syringe was used to infiltrate the transformed bacteria into the abaxial sides of *N. benthamiana* leaves. *Agrobacterium* strain GV3101 transformed with an empty vectors expressing only HA-tag (for overexpression experiment) or pdk intron (for silencing experiment) without PVX:GFP were used as negative controls. For positive control, *N. benthamiana* plants agro-infiltrated with an empty vector followed by infiltration of agrobacteria containing PVX:GFP on the same leaves were used. Experiments for overexpression and silencing were performed with three biological replicates with at least three individual plants for each experiment.

### Quantification of PVX replication in protoplasts

To quantify PVX replication, we transiently overexpressed or silenced the target gene. In the case of overexpression, PVX was challenge-inoculated on the infiltrated leaves at 1 day after infiltration. In the case of gene silencing, PVX was challenge-inoculated on the infiltrated leaves at 4 days after infiltration. At 12 h after PVX challenge inoculation, protoplasts were extracted from the inoculated leaves according to previous reports^52^,^53^. The extracted protoplasts were cultured in Aoki’s salt medium at 25 °C for 2 days. The total RNA of the cultured protoplasts was extracted as previously described^26^. PVX replication in protoplasts was quantified by real-time RT-PCR using a CFX384 real-time PCR detection system (Bio-Rad, Hercules, USA). The PCR results were normalized with actin and ubiquitin genes.

### Time-course gene expression analysis using real-time RT-PCR

For time-course expression analysis of the target host genes and PVX, we performed real-time RT-PCR using gene-specific primers (Supplementary Table S1). Plant samples were harvested from the leaves that each three viruses inoculated at each time point for 1 week. Three biological replicates were used for this experiment and conducted three times. PVX, CMV, and PepMoV were mechanically inoculated with carborundum.

### Subcellular localization of the two host genes

The full-length cDNAs of the two host genes were individually cloned into the pENTR vector. Each pENTR vector was cloned into pSITE-2CA^54^ containing GFP by LR reaction according to the manufacturer’s instructions (Invitrogen). Cellular markers tagged with RFP were co-infiltrated with vectors expressing GFP-tagged host protein or empty vector expressing free GFP (pSITE-2CA). Two days after agroinfiltration, GFP as well as RFP were observed with a fluorescence microscope (Carl Zeiss, Oberkochen, Germany). We carried out subcellular localization experiments with three independent biological replicates.

## Additional Information

**How to cite this article**: Choi, H. *et al*. Two homologous host proteins interact with potato virus X RNAs and CPs and affect viral replication and movement. *Sci. Rep.* **6**, 28743; doi: 10.1038/srep28743 (2016).

## Supplementary Material

Supplementary Information
