# Supplementary material for: Two homologous host proteins interact with potato virus X RNAs and CPs and affect viral replication and movement
Source: Sci Rep. 2016 Jun 29;6:28743. doi: 10.1038/srep28743 (PMC4926161; doi:10.1038/srep28743)
Supplement: Supplementary Information [file srep28743-s1.pdf]

Supplementary information

Two homologous host proteins interact with *Potato virus X* RNAs and CPs and affect viral replication and movement

Hoseong Choi[a,b,c], Won Kyong Cho[a], and Kook-Hyung Kim[a,b,c,*]

[a]Department of Agricultural Biotechnology, Seoul National University, Seoul, Korea

[b]Research Institute of Agriculture and Life Sciences, Seoul National University, Seoul, Korea

[c]Plant Genomics and Breeding Institute, Seoul National University, Seoul, Korea

Supplementary Figure S1. Interaction of PVX RNA structures with two host proteins. Recombinant NbCPIP2a and NbCPIP2b proteins prepared from *E. coli* were loaded on the gel. Four probes, including SL1(+), SL1(-), 3'SL, and non-viral sequences, were transcribed using T7 RNA polymerase with P^{32} labelled isotope. Arrows indicate the shifted RNA-protein complex. R indicates the well in which only probe was loaded.

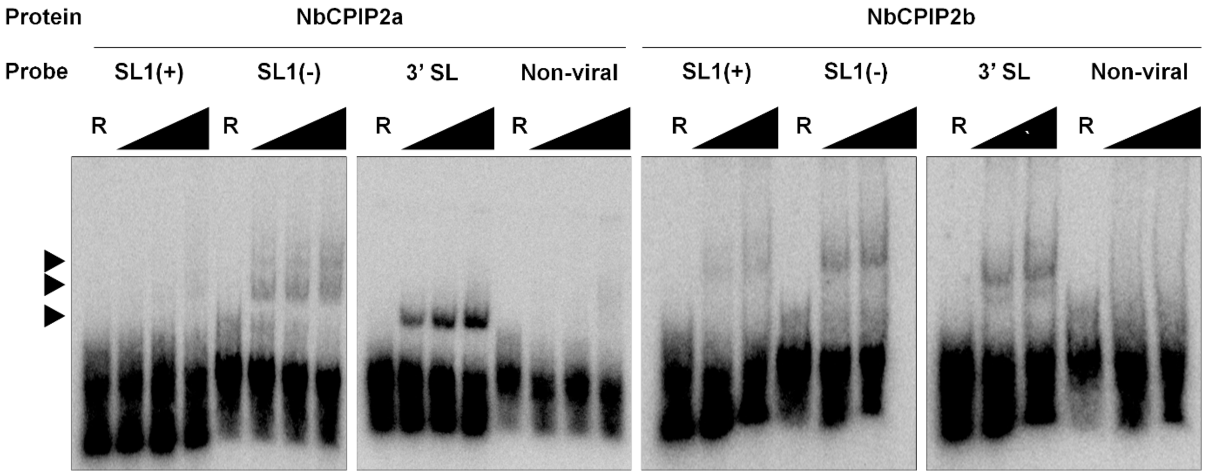

Supplementary Figure S2. Interaction of (A) NbCPIP2a and (B) NbCPIP2b with the CPs of PepMoV, SMV, PVA, PVY, TuMV, and ZYMV as determined by BiFC.

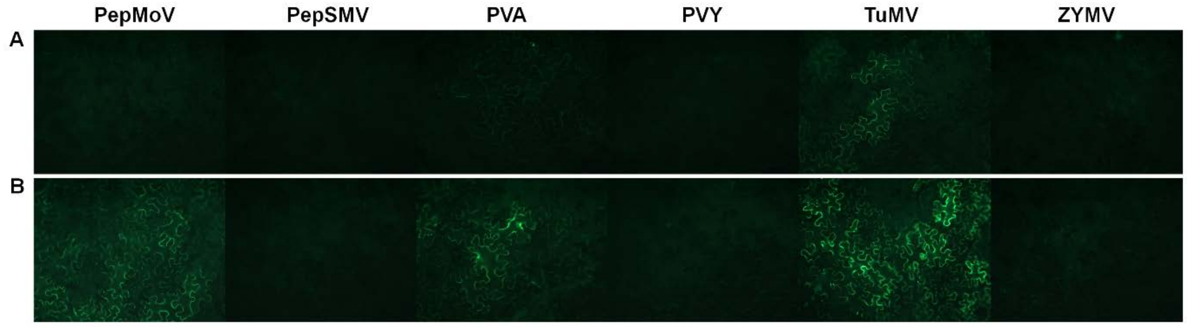

Supplementary Table S1. List of primers used in this study.

Primer name	Primer sequence	Purpose
NbCPIP_GW_F	CACCATGGGACTTGATTACTAT	Cloning
NbCPIP_GW_R	TTAGTCAGAGTTCCTGCAC	
NbCPIP_pET_F	CGGGATCCATGGGACTTGATTACTATA	Recombinant expression
NbCPIP_pET_R	GGAATTCTTAGTCAGAGTTCCTGCAC	
PVX_SL1(+)_F	CAAGCTTTAATACGACTCACTATAGGGCAACCAAACCCACCAC	EMSA
PVX_SL1(+)_R	GCTGCAGTTGGTAAACCTCGCGC	
PVX_SL1(-)_F	CAAGCTTTAATACGACTCACTATAGGGTTGGTAAACCTCGCGC	
PVX_SL1(-)_R	GCTGCAGCAACCAAACCCACCAC	
PVX_3SL_F	CAAGCTTTAATACGACTCACTATAGGGCTACGTCTACATAACCGACGC	
PVX_3SL_R	GCTGCAGTTTATTTATATTATTCATACAATC	
PVX_RdRP_GW_F	CACCATGGCCAAAGTGCGCGAGG	BiFC
PVX_RdRP_GW_R	TTAAAGAAAGTTTCTGAGGCG	
PVX_TGB1_GW_F	CACCATGGAATATTCTCATCATTAGTTTG	
PVX_TGB1_GW_R	CTATGTTCCTGCGCGGACAT	
PVX_TGB2_GW_F	CACCATGTCCGCGCAGGAACATAGAC	
PVX_TGB2_GW_R	CTAATGACTGCTATGATTGTTACCACAAG	
PVX_TGB3_GW_F	CACCATGGAAGTAAATACATATCTCAACGCAATC	
PVX_TGB3_GW_R	TCAATGGAAACTTAACCGTTCAACG	
PVX_CP_GW_F	CACATGTCAGCACCAGCTAGCACAA	
PVX_CP_GW_R	TTATGGTGGTGGGAGAGTGACA	
SMV_CP_F	CACCATGTCAGGTAAGGAGAAGGAAGGAG	
SMV_CP_R	TTACTGCTGTGGACCCATGCC	
NbCPIP2a_RNAi_F	GACGACAAGACCCTGTCATCTTAACTTAATCCGAATATT	RNAi-structure
NbCPIP2a_RNAi_R	GAGGAGAAGAGCCCTTATCTGGATGCCATTTCATCG	

NbCPIP2b_RNAi_F	GACGACAAGACCCTGTCATCTTAAACAAATCCGAATA	
NbCPIP2b_RNAi_R	GAGGAGAAGAGCCCTTCTGGATGCCATTTCATGG	
NbCPIP_RT_F	TTTTTCGGTGGATCGGATAG	Real-time qRT-PCR
NbCPIP_RT_R	CTTCCAGCCTGGTTTGATGT	
NbActin_F	CCAGGTATTGCTGATAGAATGAG	
NbActin_R	CTGAGGGAAGCCAAGATAGAG	
NbUbi3_F	GCCGACTACAACATCCAGAAGG	
NbUbi3_R	TGCAACACAGCGAGCTTAACC	
